# Transcutol^®^ P Containing SLNs for Improving 8-Methoxypsoralen Skin Delivery

**DOI:** 10.3390/pharmaceutics12100973

**Published:** 2020-10-15

**Authors:** Giulia Pitzanti, Antonella Rosa, Mariella Nieddu, Donatella Valenti, Rosa Pireddu, Francesco Lai, Maria Cristina Cardia, Anna Maria Fadda, Chiara Sinico

**Affiliations:** 1 Department of Life and Environmental Sciences, Unit of Drug Sciences, University of Cagliari, 09124 Cagliari, Italy; giulia.pitzanti@unica.it (G.P.); valenti@unica.it (D.V.); rosapireddu@unica.it (R.P.); frlai@unica.it (F.L.); cardiamr@unica.it (M.C.C.); mfadda@unica.it (A.M.F.); 2 Department of Biomedical Sciences, University of Cagliari, Cittadella Universitaria, SS 554, Km 4.5, 09042 Monserrato, Cagliari, Italy; anrosa@unica.it (A.R.); mnieddu@unica.it (M.N.)

**Keywords:** 8-MOP, diethylene glycol monoethyl ether, solid lipid nanoparticles, topical PUVA, topical delivery, psoriasis

## Abstract

Topical psoralens plus ultraviolet A radiation (PUVA) therapy consists in the topical application of 8-methoxypsoralen (8-MOP) followed by the skin irradiation with ultraviolet A radiation. The employment of classical 8-MOP vehicles in topical PUVA therapy is associated with poor skin deposition and weak skin permeability of psoralens, thus requiring frequent drug administration. The aim of the present work was to formulate solid lipid nanoparticles (SLNs) able to increase the skin permeation of 8-MOP. For this purpose, the penetration enhancer Transcutol^®^ P (TRC) was added to the SLN formulation. SLNs were characterized with respect to size, polydispersity index, zeta potential, entrapment efficiency, morphology, stability, and biocompatibility. Finally, 8-MOP skin diffusion and distribution within the skin layers was investigated using Franz cells and newborn pig skin. Freshly prepared nanoparticles showed spherical shape, mean diameters ranging between 120 and 133 nm, a fairly narrow size distribution, highly negative ζ potential values, and high entrapment efficiency. Empty and loaded formulations were almost stable over 30 days. In vitro penetration and permeation studies demonstrated a greater 8-MOP accumulation in each skin layer after SLN TRC 2% and TRC 4% application than that after SLN TRC 0% application. Finally, the results of experiments on 3T3 fibroblasts showed that the incorporation of TRC into SLNs could enhance the cellular uptake of nanoparticles, but it did not increase their cytotoxicity.

## 1. Introduction

Psoriasis is a chronic autoimmune inflammatory skin disease characterized by well-defined thickened and inflamed skin lesions (named plaques) overlaid by scales [1]. It has a bimodal age of onset (16 to 22 and 57 to 60 years) and affects men and women identically [2].

The increased knowledge of the pathophysiology of psoriasis has resulted in several new effective therapeutic options to alleviate the symptoms, targeting different extracellular and intracellular immune processes [3]. Treatment methods are commonly categorized into topical therapy, phototherapy, and systemic therapy [4].

PUVA, photochemotherapy with psoralens, and long-wavelength ultraviolet (UVA) radiation is one of the Food and Drug Administration-approved therapies for an effective clinical management of psoriasis [3]. 8-methoxypsoralen (8-MOP) is the most employed psoralen in PUVA [5,6,7,8,9]. 8-MOP, also known as methoxsalen or xanthotoxin, is a natural psoralen belonging to the furanocoumarin class. Its photosensitizing ability is exploited in the PUVA therapy [10,11]. Indeed, following oral administration (systemic PUVA) or topical application (topical PUVA), the psoralen intercalates between DNA base pairs, keeping its inactive status in the absence of ultraviolet radiation. The exposure to UVA light activates the photosensitising agent, which forms cyclobutane monoadducts with pyrimidine bases. When a second photon of energy is absorbed, psoralen forms cross-linkages between the two strands of DNA, which inhibits DNA replication and causes cell cycle arrest [7,9,12,13]. Moreover, it may increase pigmentation by improving both the activity of tyrosinase and the synthesis of melanin in the healthy melanocytes that are close to the damaged cells [8,13].

In systemic PUVA, a crystalline form of 8-MOP in the dosage of 0.6 mg/kg is commonly administered 2 h before the patient is irradiated with UVA. Alternatively, an encapsulated form at a dose of 0.4 mg/kg is administered 90 min before irradiation [13]. However, the oral administration of 8-MOP is associated with gastrointestinal side effects and with the risk of severe complications, e.g., photocarcinogenesis and glaucoma. 8-MOP gastrointestinal absorption is variable, with evident inter-individual differences in peak blood concentrations, which rapidly decrease due to a strong first-pass effect through the liver. For these reasons, topical PUVA therapy has been developed as a more safe and effective treatment, which would avoid the systemic PUVA adverse effects [8]. In topical PUVA, an emollient base containing 0.1% 8-MOP is commonly employed. It should be applied 30 min before exposure to UVA starting at a dose of 0.25 to 0.5 J/cm^2^ (Joule/cm^2^) and increasing the dose up to 0.5 J/cm^2^ depending on the patient’s tolerance [12]. Unfortunately, classical 8-MOP vehicles, such as creams, gels, and lotions, necessitate frequent drug administration due to their poor skin deposition and weak skin permeability of psoralens, which in turn leads to adverse reactions [3]. In order to overcome the drawbacks of 8-MOP conventional topical formulations, there is a need to develop dermatological formulation using novel drug delivery systems.

Solid lipid nanoparticles (SLNs) are considered a particularly useful nanocarrier for the dermal administration of lipophilic drug like psoralens. Indeed, the small particle sizes, and thus their high surface area, ensures close contact and adhesion to the stratum corneum. Moreover, SLNs form a thick lipid film on the skin surface, which induces skin hydration by an occlusive action [14,15].

The aim of this study was to develop SLNs with increased skin permeation of 8-MOP. In order to further enhance 8-MOP penetration through the skin, a penetration enhancer, namely Transcutol^®^ P (TRC), was used. TRC (diethylene glycol monoethylether) is a well-known and efficacious permeation enhancer, non-toxic and highly compatible with biological tissues, soluble both in water and in oil (KO/A = 0.7), and employed in several pharmaceutical formulations. It works as a potent solubilizing agent, which swells the intercellular lipid matrix of the stratum corneum, thus allowing drug to penetrate into the reversibly modified skin barrier [16].

The SLNs were produced by a hot homogenization technique followed by ultrasonication and were characterized by their size distribution, polydispersity index, entrapment efficiency, morphology, and stability. We also present our findings concerning the effects of these vehicles on both the diffusion of the drug through the skin and its distribution within the skin layers. Finally, in vitro studies using murine fibroblasts were performed to investigate 8-MOP SLN biocompatibility.

## 2. Materials and Methods

### 2.1. Materials

8-MOP and Pluronic F68 (Poloxamer 188) were purchased from Sigma Aldrich (Milan, Italy). Compritol^®^ 888 ATO and Transcutol^®^ P (TRC) were kindly supplied by Gattefossè (Lyon, France). Cell culture materials were purchased from Invitrogen (Milan, Italy). Cholesterol, standards of fatty acids, 3-(4,5-dimethylthiazol-2-yl)-2,5-diphenyltetrazolium bromide (MTT), 1,2-dipalmitoyl-*sn*-glycero-3-phosphocholine (PC 16:0/16:0), 1,2-dioleoyl-sn-glycero-3-phosphocholine (PC 18:1/18:1), 1-palmitoyl-2-oleoyl-*sn*-glycero-3-phosphocholine (PC 16:0/18:1), 1-oleoyl-2-palmitoyl-sn-glycero-3-phosphocholine (PC 18:1/16:0), 2-linoleoyl-1-palmitoyl-sn-glycero-3-phosphocholine (PC 16:0/18:2), 2-arachidonoyl-1-palmitoyl-sn-glycero-3-phosphocholine (PC 16:0/20:4), 1,2-dilinoleoyl-sn-glycero-3-phosphocholine (PC 18:2/18:2), and 1,2-dieicosapentaenoyl-sn-glycero-3-phosphocholine (PC 20:5/20:5) were purchased from Sigma–Aldrich (Milan, Italy). All the other chemicals used in this study were of analytical grade.

### 2.2. SLN Preparation

All empty and 8-MOP-loaded SLNs containing Transcutol^®^ P or not were prepared by a hot homogenization technique followed by ultrasonication. Briefly, 8-MOP was dissolved in the melted Compritol 888 ATO. A hot aqueous solution of Poloxamer 188 and Transcutol^®^ P (when present in the formulation, SLN TRC 2% and SLN TRC 4%), heated at the same temperature of the lipid phase, was dispersed into the drug-containing melt. The pre-emulsion was then homogenized using a high share homogenizer (Ultra Turrax T25 basic) for 1 min at 6500 rpm and then sonicated for 4 min with a Soniprep 150 (MSE Crowley, London, UK) thermostated at 85 °C. The obtained hot O/W nanoemulsion was cooled down to room temperature and SLNs were formed by the lipid re-crystallization. Additionally, empty control SLNs were prepared using the same procedure and the same surfactant concentration of loaded formulations. The SLN composition is reported in Table 1.

### 2.3. Characterization of Solid Lipid Nanoparticles

#### 2.3.1. Encapsulation Efficiency

The entrapment efficiency (EE%), which represents the percentage of encapsulated drug within nanoparticles with respect to the amount used during preparation, was measured by an indirect method. Briefly, 500 µL of 8MOP-SLN dispersions were placed in the upper chamber of an Amicon^®^ Ultra-0.5 (30 kDa) and centrifuged at 15,000 rpm for 30 min using a cooling centrifuge (Scilogex mod. D3024R, Rocky Hill, CT, USA) to separate the lipid and aqueous phase. The unentrapped drug collected in the filtrate in the lower chamber was assayed by HPLC after being properly diluted with methanol. The entrapment efficiency (EE%) was calculated according to the following equation:

(1) $$\mathrm{EE}\%=\frac{\left(\mathrm{Amount}\;\mathrm{of}\;\mathrm{drug}\;\mathrm{added}-\mathrm{Amount}\;\mathrm{of}\;\mathrm{free}\;\mathrm{drug}\right)}{\left(\mathrm{Amount}\;\mathrm{of}\;\mathrm{drug}\;\mathrm{added}\right)}\times 100$$

#### 2.3.2. Particle Size and Zeta Potential Analysis

The average diameter and polydispersity index (PDI, a measure of the size distribution width) of the SLN were determined by photon correlation spectroscopy (PCS) using a Zetasizer nano (Malvern Instruments, Worcestershire, UK). Samples were backscattered by a helium–neon laser (633 nm) at an angle of 173° and a constant temperature of 25 °C.

Zeta potential was estimated using the Zetasizer nano by means of the M3-PALS (phase analysis light scattering) technique. Just before the analysis, the SLN were diluted with bidistilled water. All the measurements were made in triplicate.

A medium-term stability study of SLN stored at 25 ± 1 °C was performed by monitoring the average size, polydispersity, and surface charge for 30 days.

#### 2.3.3. Transmission Electron Microscopy (TEM)

The morphology of 8-MOP SLNs was visualized using a transmission electron microscopy (TEM) Jeol JEM 1400 Plus (Jeol, Milan, Italy) operating at 120 kV. A drop of SLN dispersion and an equal volume of an aqueous 1% phosphotungstic acid solution were adsorbed on the surface of a copper grid and dried at room temperature. 

### 2.4. 8-MOP HPLC Quantification

8-MOP quantitative analysis was performed using a chromatograph Alliance 2690 (Waters, Milan, Italy) equipped with a multi λ fluorescence detector and a computer integrating software (Empower 3) at 317 and 445 nm as the excitation wavelength and emission wavelength, respectively. The column was a X Terra RP18 (3.5 μm, 4.6 mm × 100 mm, Waters), and the mobile phase was a mixture of water:methanol:acetonitrile (40:40:20, *v*/*v*) eluted at a flow rate of 0.5 mL/min. A standard calibration curve was built up by using standard solutions. Calibration graphs were plotted according to the linear regression analysis, which gave a correlation coefficient value (R2) of 0.999.

### 2.5. In Vitro Release Experiments

In vitro release experiments of 8-MOP from the SLNs under investigation were carried out using Franz-type diffusion cells (LGA, Berkeley, CA, USA). Cellulose membranes were soaked in water for 1 h at room temperature. After 1h, they were mounted in Franz-type diffusion cells between the donor and the receptor compartment. A hydroalcoholic solution composed of water:ethanol in the ratio of 50:50 (*v*/*v*) was used as release medium to ensure pseudo-sink conditions by increasing the active compound solubility in the receiving phase. The release medium was constantly stirred using a magnetic stirrer and thermostated at 35 °C throughout the study. In total, 200 µL of each formulation were applied on the membrane surface under non occlusive conditions and the experiments were run for 24 h. At regular time intervals, 200 µL of the receptor medium were withdrawn and replaced with an equivalent volume of pre-thermostated (35 °C) fresh medium. The 8-methoxypsoralen concentration in the receptor medium samples was determined by HPLC. Each experiment was performed in triplicate.

### 2.6. In Vitro Skin Penetration and Permeation Studies

Experiments were performed non-occlusively using vertical diffusion Franz cells with an effective diffusion area of 0.785 cm^2^, using hybrid Goland-Pietrain newborn pig skin. The pigs (~1.2 kg) died of natural causes a few hours after birth, and they were provided to us by a local slaughterhouse. The pig hair was shaved and the skin was excised using a surgical scalpel and the subcutaneous fat was removed. The skin was carefully observed to visualize any wounds, punctures, holes, bleeding, or skin disease. The skin was cut into squares of 3 × 3cm^2^, randomized, and stored −80 °C until the experiments. Then, 24 h before the experiments, the skin samples were hydrated in physiological solution (0.9%, *w*/*v* of NaCl) at 25 °C and inserted between the donor and receptor compartments of the Franz cells, with the epidermis side fronting the donor cell. The receptor cell was filled with 5.5 mL of hydroalcoholic solution (phosphate-buffered saline solution/ethanol 1/1, *w*/*w*), which was constantly stirred with a small magnetic bar and thermostated at 37 ± 1 °C throughout the experiments to reach the physiological skin temperature (i.e., 32 ± 1 °C). In total, 100 µL of either 8-MOP-loaded SLNs were applied on the stratum corneum (*n* = 6 skin pieces per tested formulation). At 2 h intervals up to 8 h, the receiving medium was withdrawn, analyzed by HPLC to measure the 8-MOP content, and replaced with an equivalent volume of hydroalcoholic mixture to ensure sink conditions. After 8 h, the skin surface was washed 3 times with 1 mL of distilled water, and then dried with filter paper. The stratum corneum was removed by stripping with an adhesive tape Tesa^®^ AG (Hamburg, Germany). A piece of the adhesive tape was strongly pressed on the stratum corneum and rapidly pulled off with one stroke. The stripping procedure was repeated 10 times consecutively. The epidermis was separated from the dermis with a surgical scalpel. The tape strips, epidermis, and dermis were cut and placed each in a flask with methanol and sonicated (Soniprep 150, MSE Crowley, London, UK) for 4 min for 8-MOP extraction. The tapes and tissue suspensions were centrifuged for 10 min at 10,000 rpm, and the supernatant was filtered and assayed for 8-MOP content by HPLC.

### 2.7. Cell Culture

3T3 mouse fibroblasts, obtained from ATCC collection, were grown in Dulbecco’s modified Eagle’s medium (DMEM) (Invitrogen, Milan, Italy) enriched with fetal bovine serum (10% *v*/*v*) and penicillin (100 U mL^−1^)-streptomycin (100 μg mL^−1^) at 37 °C in a 5% CO_2_ incubator.

### 2.8. Cytotoxicity Assessment, MTT Assay

SLNs’ cytotoxic effect was evaluated in 3T3 fibroblasts by the MTT (3-(4,5-dimethylthiazol-2-yl)-2,5-diphenyl tetrazolium bromide) assay as previously reported [17]. 3T3 cells, 2 days after seeding (at a density of 3 × 10^4^ cells/well) in 96-well plates, were treated with different concentrations (1.25, 2.5, and 5 µL/mL) of SLNs formulations in 100 µL of complete medium. After 24 h of incubation at 37 °C, cells were subjected to the viability MTT test and color development was measured at 570 nm as previously reported [17]. SLNs’ effects on 3T3 cell growth are shown as the percent of cell viability in comparison with non-treated control cells. The cytotoxicity of pure 8-MOP at the dose of 5 µg/mL (from a 1 mg/mL DMSO solution) was also evaluated in 3T3 cells (24 h of incubation) for comparison. Evaluation of the cell morphology after 24 h of incubation with different SLNs was also performed by microscopic observation with a ZOE™ Fluorescent Cell Imager (Bio-Rad Laboratories, Hercules, CA, USA).

### 2.9. Lipid Profile Modulation in 3T3 Fibroblasts

3T3 fibroblasts, 2 days post-seeding (at a density of about 10^6^ cells/10 mL of complete medium) in Petri dishes, were treated with 5 µL/mL of SLNs formulations in fresh medium. After 24 h of incubation at 37 °C, the cells were washed with PBS and scraped. After centrifugation (1200× *g* at 4 °C for 5 min), cell pellets were separated from supernatants and used for the extraction and analyses of lipid compounds.

### 2.10. 3T3 Cell Lipid Extraction and Analysis

Total lipids were extracted from 3T3 cell pellets using the mixture CHCl_3_/MeOH/H_2_O 2:1:1 as previously reported [18,19]. Dried aliquots of the CHCl_3_ fraction, from each cell sample, dissolved in MeOH, were injected into the HPLC system for the direct analysis of lipid components (phospholipids, PL, and free cholesterol, FC) [18,19]. Another aliquot of dried CHCl_3_ fractions, dissolved in EtOH, was subjected to mild saponification [19]. The saponifiable phase with free fatty acids was collected and a portion of the dried residue was dissolved in CH_3_CN/0.14% CH_3_COOH (*v*/*v*). Analyses of lipid compounds were carried out with a 1100 HPLC equipped with a DAD and an Agilent 1100 HPLC-DAD/1260 ELSD system, equipped with an Inertsil ODS-2 column, for the direct analysis of lipid components (phospholipids, PL, and free cholesterol, FC) with MeOH as the mobile phase, at a flow rate of 2 mL/min and ELSD detection, as previously reported [18,19]. Another aliquot of dried CHCl_3_ fractions, dissolved in EtOH, was subjected to mild saponification [19]. The mixture of free fatty acids (saponifiable phase) was collected, dissolved in CH_3_CN/0.14% CH_3_COOH (*v*/*v*), and injected into the 1100 HPLC-DAD/1260 ELSD system according to the literature [19]. Unsaturated (DAD detection, 200 nm) and saturated fatty acids (ELSD detection) were separated with an XDB-C_18_ Eclipse column with the mixture CH_3_CN/H_2_O/CH_3_COOH (75/25/0.12, *v*/*v*/*v*) as the mobile phase (at a flow rate of 2.3 mL/min) as previously reported [17,19]. The Agilent OpenLAB Chromatography data system was used for the recording/integration of the chromatogram data. Lipid components were identified using standard compounds and UV spectra. Calibration curves, constructed using standard compounds, were linear (DAD) and quadratic (ELSD) for unsaturated and saturated fatty acids, respectively [19].

## 3. Results and Discussion

In the present study, a series of SLN formulations were prepared by a hot homogenization technique followed by ultrasonication and deeply characterized, as described in the experimental section. Empty and 8-MOP-loaded nanoparticles were obtained using Compritol 888 ATO as lipid matrix (SLN TRC 0%) or a combination of Compritol 888 ATO and TRC at different concentrations (2%, 4%) (SLN TRC 2%, SLN TRC 4%), while Pluronic F68 was always used as stabilizer. The SLN compositions are reported in Table 1.

**Figure 1:**
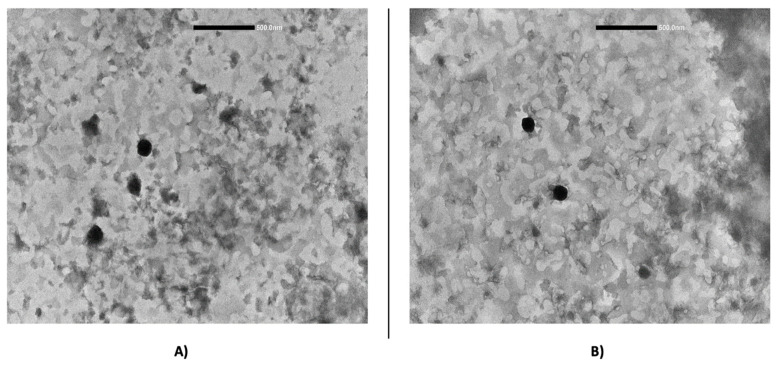
TEM micrograph of 8-MOP-loaded SLN TRC 0% (**A**) and SLN TRC 2% (**B**).

The nanoparticles’ morphology was evaluated by transmission electron microscopy (TEM), which also confirmed the formation of SLN. In Figure 1, micrographs representative of the studied samples are shown and reveal that freshly prepared nanoparticles were almost spherical in shape and with a smooth surface and there was no visible aggregation between them. The SLNs show diameters lower than the values obtained by DLS analysis. However, we would like to point out that this result is in accordance with the literature data [20,21], and can be explained by the ability of DLS to measure the hydrodynamic diameter of hydrated particles, which is also influenced by all substances adsorbed on the nanoparticle surface (hydration layer, polymer shell, or surfactants). Thus, it is always larger than the dry particle diameter obtained with TEM or SEM, which measure the geometrical size.

The characterization of the studied SLNs can be seen in Table 2, where the mean diameter, PDI, zeta potential, and EE are presented.

PCS analysis on all empty and loaded formulations was performed on the same day of preparation.

As concerns the mean diameter, it is interesting to point out that both empty and 8-MOP-loaded SLNs exhibited a mean diameter that ranged between very close values (120 and 133 nm) without any statistically significant differences in size (*p* > 0.05). These data suggest that 8-MOP incorporation does not directly influence the nanoparticle size, although they were prepared with different TRC concentrations.

The polydispersity index values for all formulations were always smaller than 0.25, thus indicating a fairly narrow size distribution. Both empty and loaded formulations showed highly negative ζ potential values ranging between −30 and −37 mV (no statistically significant difference was found for all empty and loaded SLNs).

The entrapment efficiency for all 8-MOP-loaded formulations was very high (EE% ≈ 97–99%) and independent of the TRC concentration (Table 2).

**Figure 2:**
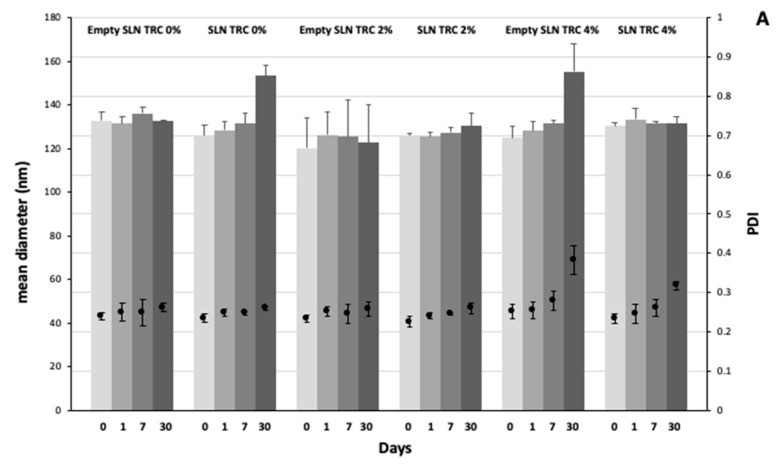

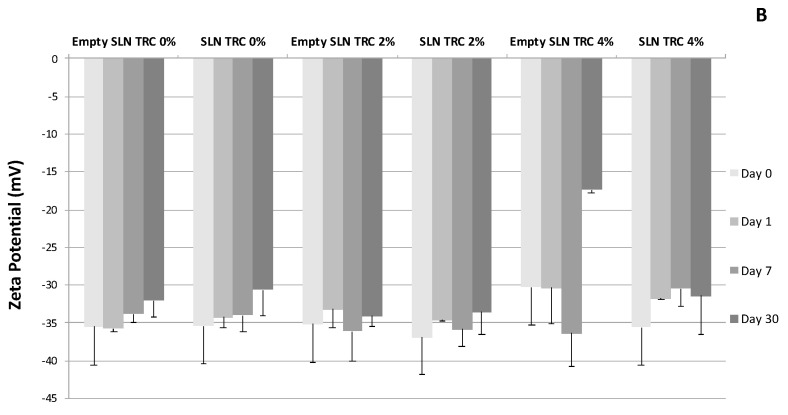
Values of mean diameter, polydispersity index (**A**), and zeta potential (**B**) of 8-MOP-loaded and -unloaded SLNs collected over 30 days of storage at 25 ± 1 °C. Mean values ± standard deviation (error bars) were reported from at least 3 independent samples.

Stability studies were performed by monitoring for 30 days variations in the size, PDI, and ζ potential of SLNs stored at 25 °C (Figure 2A,B).

As shown in Figure 2A, SLNs’ mean diameter and polydispersity index values remained constant over the studied period. Only for formulation SLN TRC 0%, and empty SLN TRC 4%, a moderate growth of the mean diameter (approximately +20% with respect of the freshly prepared formulations) was reported at day 30.

As expected, the ζ potential remained negative for all empty and loaded formulations, and almost stable in a narrow range (−30–−37 mV) over the monitored period (Figure 2B). Only the empty SLN TRC 4% formulation showed a significative ζ potential variation (−18 mV) at day 30. Since after 30 days of storage this formulation shows also an increase in size and polydispersity index, probably some aggregation phenomena occurred. Finally, the drug stability during storage was confirmed by HPLC.

Before studying the skin permeation behavior, an 8-MOP release study through a cellulose membrane was performed. Since 8-MOP is a poorly water-soluble drug, an increase of its release from SLN should result in an increase of its thermodynamic activity and, therefore, in an increase in its diffusion rate. However, the 8-MOP release rate from the studied SLNs was really slow for all the studied formulations during the first 8 h, even when using a hydroalcoholic solution as receptor fluids.

To evaluate the capability of SLNs with an increasing concentration of TRC to enhance the dermal and transdermal delivery of 8-MOP, in vitro penetration and permeation studies through newborn pig skin were carried out using Franz vertical diffusion cells. Experiments were performed in non-occlusive conditions for 8h and using the whole skin, since this model enabled us to measure both drug skin flux as well as drug accumulation in the skin strata. As for skin choice, previous studies with newborn pig skin demonstrated that this animal model provides reliable information to predict drug permeation through human skin. Indeed, newborn pig stratum corneum has a lipid composition and thickness very similar to that of human [22,23].

**Figure 3:**
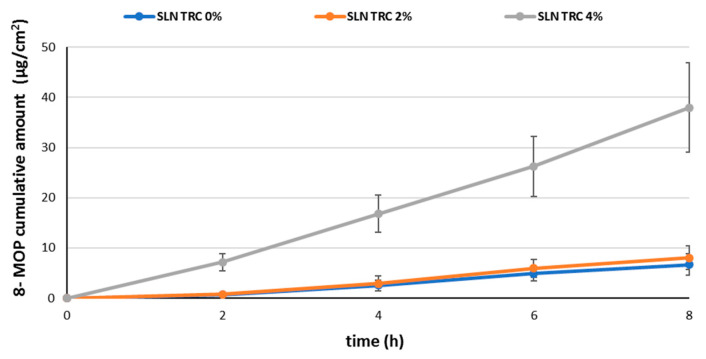
Cumulative amount of permeated 8-MOP per area against time.

In Figure 3, the amount of permeated 8-MOP per area is plotted against time. Examination of the graphs shows that all the studied SLNs reached steady-state flux but after different lag times. In a previous work, Fang et al. [14] investigated the skin permeation of 8-MOP from an aqueous suspension, a lipid emulsion, and nanoparticulate lipid systems (SLN and NLC). Compared to the lipid emulsion and aqueous control, enhanced psoralen permeation was achieved with the SLN and NLC. In particular, the flux was found to be higher for both NLC vehicles (21.0 µg cm^−2^ h^−1^), SLN (14.5 µg cm^−2^ h^−1^), and aqueous suspension (13.1 µg cm^−2^ h^−1^), and lower for lipid emulsion (8.3 µg cm^−2^ h^−1^). In this study, the SLN TRC 0% and SLN TRC 2% formulations provided a lower flux (1.00 and 0.83 μg cm ^−2^ h^−1^ respectively) than the formulations studied by Fang et al. [14]. Indeed, after 8 h, only 5% (SLN TRC 0%) and 6% (SLN TRC 2%) of the drug was released through the skin. This aspect is of great interest because it suggests that the drug is retained in the skin for a long time and systemic effects are minimized. By contrast, the mean amount of drug permeated after 8 h of experiment from SLN TRC 4% was 37.4 μg, with a steady-state flux of 4.75 μg cm^−2^ h^−1^. This is not surprising, given the different concentrations of penetration enhancers between the tested SLNs. In these experiments, we did not use control formulations, such as emulsions or other lipidic nanocarriers, since 8-MOP solubility in different vehicles is different and, therefore, also its thermodynamic activity would be different. As a consequence, comparisons between in vitro skin permeation results would be unreliable [24].

**Figure 4:**
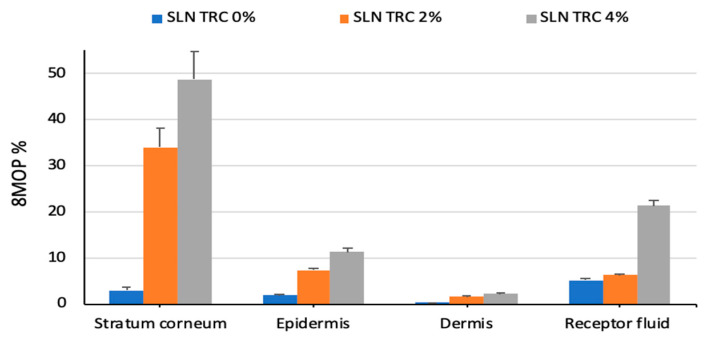
In vitro skin penetration and permeation studies: amount of 8-MOP accumulated in the skin layers (stratum corneum; epidermis; dermis) and receptor fluid after 8 h of non-occlusive application of the SLN. Each value is the mean ± standard deviation of six experimental determinations.

Finally, the % of 8-MOP accumulated in the different skin layers (stratum corneum, epidermis, and dermis) and permeation through the whole skin after application of SLN with or without TRC is reported in Figure 4. In accordance with the results obtained with other lipidic nanocarriers [9], all the SLNs that we developed showed higher drug accumulation in the stratum corneum as compared to viable epidermis and dermis, since these strata have higher hydrophilic characteristics than thee stratum corneum. Therefore, lipophilic substances, such as 8-MOP, tend to accumulate in this tissue. It is worth noticing that after SLN TRC 2% and TRC 4% application, the % of the drug accumulated in each skin layer was significantly greater than that found after SLN TRC 0% application. The ability of different lipidic nanocarriers to enhance 8-MOP skin delivery has been previously reported in the literature [6,7,8,9,10,11,14,25,26,27,28]. For instance, in our group’s previous study, we incorporated 8-MOP into vesicular carriers (liposomes and niosomes) positively or negatively charged and we investigated their effect on 8-MOP diffusion through the skin and on its distribution within the skin layers. In comparison to the drug hydroalcoholic solution, all the vesicular carriers increased both the skin’s permeation and accumulation [7]. The results obtained in this work with Transcutol SLNs revealed up to around a three-fold higher skin accumulation as compared by the data obtained with vesicles.

In a very recent work, Oliveira et al. investigated 8-MOP nanoemulsions for topical treatment of skin diseases. The ex vivo permeation study showed that 8.5% of the applied 8-MOP permeated through the skin, with a flux of 1.35 μg cm^−2^ h^−1^. The skin drug retention was almost two-fold higher than a commercial cream (~23% and 14%, respectively) [10]. Once again, our formulations lead to a higher accumulation into the whole skin, reaching the maximum values for the formulation with the highest concentration of Transcutol (~62% of the applied dose).

Indeed, the penetration enhancer is able to interact with the network of keratin and lipids (free fatty acids, cholesterol, and long-chain ceramides) that makes up the stratum corneum (SC), thus reducing its barrier effect. Moreover, it has been reported that Transcutol is a hygroscopic compound that can absorb water from the skin and can change the solubility of many drugs, thus improving their skin penetration into the inner skin strata [29]. The higher 8-MOP accumulation into the skin obtained after the application of Transcutol SLNs as compared to the drug skin accumulation obtained after the application of free-Transcutol SLNs may be a consequence of the combination of the two mechanisms of action of this penetration enhancer.

The overall results of the transdermal experiments clearly suggest that the higher the TRC%, the higher the flux and the skin accumulation. Higher skin accumulation of 8-MOP obtained with Transcutol P-containing SLNs might improve the efficacy of topical PUVA in psoriasis and minimize the risk of serious adverse effects.

**Figure 5:**
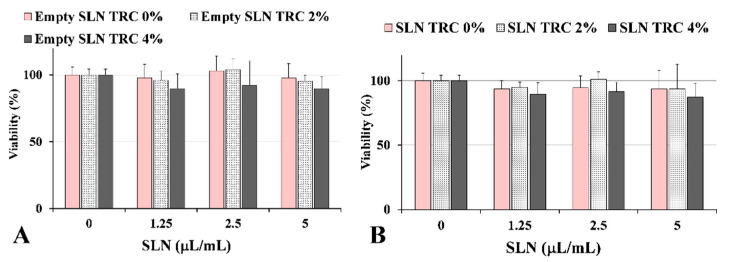
Viability (expressed as % of the control) (MTT assay) measured in control 3T3 fibroblasts (0) and cells treated for 24 h with three aliquots (1.25, 2.5, 5 µL/mL) of unloaded SLNs (empty SLN) (**A**) and 8-MOP-loaded SLNs (SLN) (**B**) with different % of TRC (0%, 2%, 4%). Data are expressed as a mean ± standard deviation (SD) of three independent experiments involving triplicate analyses for each sample (*n* = 9).

SLNs formulations were tested for cytotoxicity (MTT assay) in murine 3T3 fibroblasts. The MTT assay has been previously used to test cell viability and mitochondrial activity after treatment with biocompatible nanoparticles [19,30]. Figure 5 shows the viability, expressed as % of the control, induced in 3T3 fibroblasts after 24 h of incubation in the presence of different concentrations (1.25, 2.5, and 5 µL/mL) of unloaded SLNs (empty SLN) (Figure 5A) and 8-MOP loaded SLNs (SLN) (Figure 5B) with different % of TRC (0%, 2%, 4%). The treatment with all formulations did not induce a significant reduction in cell viability in the tested range with respect to the control cells. Pure 8-MOP, added to cells at the maximal dose present in the tested formulations was not toxic for 3T3 cells, and the cell viability was 96% (data not shown).

**Figure 6:**
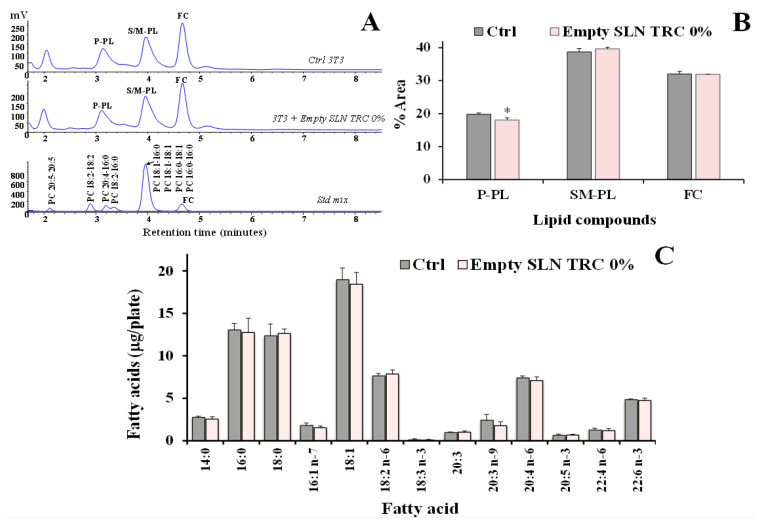
(**A**) Chromatographic profile, obtained by HPLC-ELSD analysis, of lipid compounds (saturated/monounsaturated phospholipids, S/M–PL; polyunsaturated phospholipids, P-PL; free cholesterol, FC) measured in control 3T3 fibroblasts (Ctrl) and cells treated for 24 h with unloaded SLN without TRC (empty SLN TRC 0%) (**A**); the chromatographic region for each lipid class was assigned by using standard mixtures of saturated/monounsaturated (mix PL: PC 16:0/16:0, PC 18:1/18:1, PC 16:0/18:1, PC 18:1/16:0, ECN 32) and polyunsaturated phosphatidylcholines (PC 16:0/18:2, ECN 30, PC 16:0/20:4, PC 18:2/18:2, ECN 28, PC 20:5/20:5, ECN 20). Values of PL and FC (% area) (**B**) and the main fatty acids (expressed as µg/plate) (**C**) measured in control 3T3 and fibroblasts treated with empty SLN TRC 0% (24 h). Two independent experiments and tree replicates for each condition are performed and data are presented as mean ± SD (*n* = 6); * = *p* < 0.05 versus respective Ctrl (Student’s unpaired t test with Welch’s correction).

A study was undertaken to investigate the impact of SLNs on the 3T3 lipid profile as a marker of the cellular internalization of lipid nanoparticles. Figure 6A shows the chromatographic profile, obtained by HPLC-ELSD analysis, of polar lipid compounds (saturated/monounsaturated phospholipids, S/M–PL; polyunsaturated phospholipids, P-PL; free cholesterol, FC) measured in control 3T3 fibroblasts and cells treated for 24 h with unloaded SLNs without TRC (empty SLN TRC 0%) (5 µL/mL). Standard mixtures of saturated, monounsaturated, and polyunsaturated phosphatidylcholines and FC were used for the assignment of the chromatographic region for each lipid class [19]. The equivalent carbon number ECN = CN − 2*n* (CN is the number of acyl group carbons; *n* the number of double bonds) was used for the chromatographic separation of lipid components [19]. The polar lipid profile of control 3T3 cells was characterized by a peak of FC and two main peaks of PL, corresponding to S/M-PL and P-PL. 3T3 cells treated with empty SLN TRC 0% showed a slight change in the lipid profile, with a significant decrease in the % peak area of P-PL (*p* < 0.05 versus untreated cells) and a small increase in the % of S/M-PL (Figure 6A). Figure 6C shows the values of the main fatty acids (expressed as µg/plate) measured in control 3T3 and fibroblasts treated with empty SLN TRC 0% (24 h). Control 3T3 showed a lipid composition characterized by a high level of 18:1 isomers (18.96 ± 1.40 µg/plate; mainly oleic acid 18:1 *n* − 9), palmitic acid (16:0), stearic acid (18:0), arachidonic acid (20:4 *n* − 6), and linoleic acid (18:2 *n* − 6). The incubation (for 24 h) of 3T3 cells with empty SLN TRC 0% did not induce evident changes in fatty acid levels, with treated cells showing a profile similar to that of control cells.

**Figure 7:**
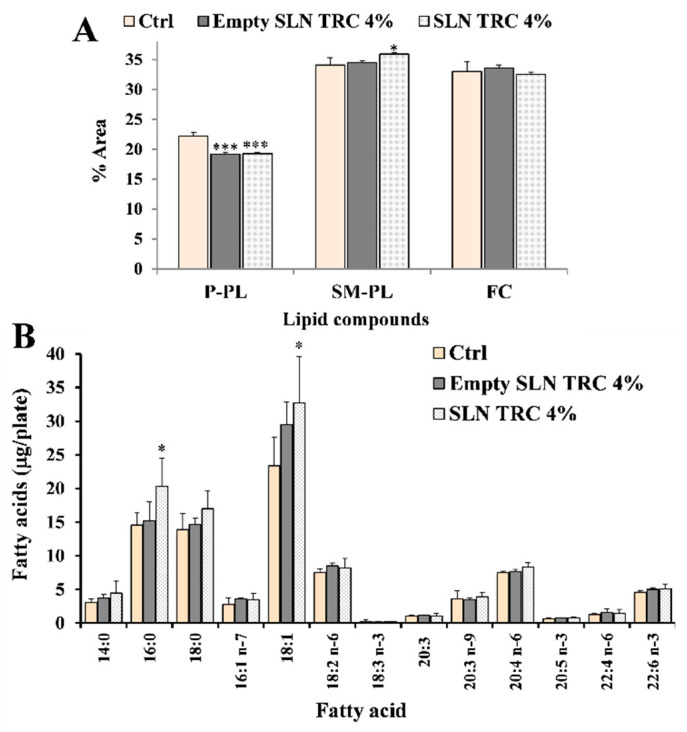
Values of lipid compounds (saturated/monounsaturated phospholipids, S/M–PL; polyunsaturated phospholipids, P-PL; free cholesterol, FC) (% area) (**A**) and the main fatty acids (expressed as µg/plate) (**B**) measured in control 3T3 (Ctrl) and fibroblasts treated for 24 h with unloaded (empty SLN TRC 4%) and 8-MOP-loaded (SLN TRC 4%) SLN with 4% TRC. Two independent experiments and tree replicates for each condition are performed and data are presented as mean ± SD (*n* = 6); *** = *p* < 0.001; * = *p* < 0.05 versus Ctrl (One–way ANOVA followed by the Bonferroni Multiple Comparisons Test).

Figure 7 shows the values of polar lipid compounds (S/M–PL, P-PL, FC) (% area) (Figure 7A) and main fatty acids (µg/plate) (Figure 7B) measured in 3T3 fibroblasts treated for 24 h with empty and 8-MOP-loaded SLNs with 4% of TRC (empty SLN TRC 4% and SLN TRC 4%) compared to the respective control cells. Both formulations (at the dose of 5 µL/mL) induced in 3T3 cells a change in the profile of polar lipids, in particular a marked (*p* < 0.001 versus untreated cells) decrease was observed in the % of peak corresponding to P-PL with respect to the other peaks, coupled to an increase in the % of S/M-PL (Figure 7A), with the latter effect more marked in cells treated with SLN TRC 4%. The 24 h treatment of fibroblasts with both formulations also affected the cell fatty acid profile. A remarkable increase in the cell levels of 18:1 *n* − 9 (*p* < 0.05 versus untreated cells), 16:0 (*p* < 0.05), and 18:0 was detected in fibroblasts incubated with SLN TRC 4%, while the treatment did not seem to affect the levels of the other unsaturated fatty acids, with respect to control cells. The fatty acid profile modulation was less marked in 3T3 cells treated with empty SLN TRC 4%, with an evident, but not significant, increase of 18:1 *n* − 9.

The modulation of the fatty acid profile was compatible with a potential intracellular uptake of the SLN formulation and the metabolism of behenic acid, the main fatty acid of Compritol^®^ 888 ATO that is constituted by a mixture of glycerol tribehenate, dibehenate, and monobehenate. A previous study reported the cellular uptakes of SLNs composed of different lipid materials and the order of cellular uptake ability was glycerol tristearate SLN > monostearin SLN > stearic acid SLN > Compritol^®^ 888 ATO SLN [31]. Our results showed that the incorporation of TRC into SLNs could enhance the cellular uptake of nanoparticles, but it did not increase their cytotoxicity. Presumably, after the addition to the culture media, SLNs with 4% of TRC were taken up by 3T3 cells by endocytosis [30], and behenic acid, derived from the lipolysis of its derivatives, was possibly metabolized/oxidized and its oxidation products were used for the synthesis of phospholipids incorporating 18:1 *n* − 9, 16:0, and 18:0.

## 4. Conclusions

Increasing 8-MOP skin penetration and accumulation can improve its efficacy in the local treatment of psoriasis. To this aim, here, we combined a nanoparticle lipid carrier, namely SLN, with a penetration enhancer (TRC) to facilitate drug diffusion through the skin barrier. The enhancement activity of different TRC concentrations was evaluated on porcine skin.

The overall results of the transdermal experiments clearly suggest that the higher the TRC %, the higher the flux and the skin accumulation of 8-MOP.

Finally, SLNs formulations were tested on fibroblasts to investigate both the cytotoxicity and their impact cell lipid profile. Outcomes showed that the incorporation of TRC into SLNs could enhance the cellular uptake of nanoparticles, but it did not increase their cytotoxicity.

Further research should be carried out in order to test the in vivo performances of our formulations in epidermal hyperproliferation simulating psoriasis-affected animal skin.

## Figures and Tables

**Table 1:** Composition of 8-MOP-loaded and -unloaded SLNs.

Formulations	Compritol 888 ATO (%wt/wt)	P188 (%wt/wt)	8-MOP (%wt/wt)	Transcutol^®^ P (%wt/wt)	Water (%wt/wt)
Empty SLN TRC 0%	4	2.2	–	–	93.8
SLN TRC 0%	4	2.2	0.1	–	93.7
Empty SLN TRC 2%	4	2.2	–	2	91.8
SLN TRC 2%	4	2.2	0.1	2	91.7
Empty SLN TRC 4%	4	2.2	–	4	89.8
SLN TRC 4%	4	2.2	0.1	4	89.7

**Table 2:** Mean diameter (MD), polydispersity index (PDI), zeta potential (ZP), and % entrapment efficiency (EE%) observed for freshly prepared 8-MOP-loaded and -unloaded SLNs using different amounts of Transcutol^®^ P. Mean values ± standard deviation, obtained from at least 3 independent samples, were reported.

Formulations	MD (nm) ± S.D.	PDI ± S.D.	ZP (mV) ± S.D.	EE% ± S.D.
Empty SLN TRC 0%	132.9 ± 3.8	0.238 ± 0.010	−35.5 ± 1.5	-
SLN TRC 0%	125.8 ± 5.2	0.234 ± 0.010	−35.4 ± 2.0	97.2 ± 0.2
Empty SLN TRC 2%	120.1 ± 13.8	0.233 ± 0.009	−35.2 ± 1.7	-
SLN TRC 2%	126.0 ± 1.0	0.225 ± 0.013	−36.9 ± 1.5	97.4 ± 0.3
Empty SLN TRC 4%	125.0 ± 5.5	0.251 ± 0.018	−30.3 ± 5.1	-
SLN TRC 4%	130.5 ± 1.2	0.234 ± 0.012	−35.6 ± 1.4	99.6 ± 0.6
